# Herpes viruses and human papilloma virus in nasal polyposis and controls^☆^

**DOI:** 10.1016/j.bjorl.2015.08.010

**Published:** 2015-09-08

**Authors:** Dimitrios Ioannidis, Vasileios A. Lachanas, Zoe Florou, John G. Bizakis, Efthymia Petinaki, Charalampos E. Skoulakis

**Affiliations:** a Microbiology Department, Medical School, University of Thessaly, Larissa, Greece; b Department of Otorhinolaryngology, University Hospital of Larissa, Larissa, Greece; c Department of Otorhinolaryngology, Medical School, University of Thessaly, Larissa, Greece

**Keywords:** Sinusitis, Nasal polyps, Viruses, Herpesviridae, Sinusite, Pólipos nasais, Herpesviridae, Papilomavírus humano

## Abstract

**Introduction:**

Chronic rhinosinusitis with nasal polyps is a multifactorial disease entity with an unclear pathogenesis. Contradictory data exist in the literature on the potential implication of viral elements in adult patients with chronic rhinosinusitis.

**Objective:**

To compare the prevalence of human herpes viruses (1–6) and Human Papilloma Virus in adult patients with chronic rhinosinusitis with nasal polyps and healthy controls.

**Methods:**

Viral DNA presence was evaluated by real-time polymerase chain reaction application to nasal polyps specimens from 91 chronic rhinosinusitis with nasal polyps patients and nasal turbinate mucosa from 38 healthy controls.

**Results:**

Epstein–Barr virus positivity was higher in nasal polyps (24/91; 26.4%) versus controls (4/38; 10.5%), but the difference did not reach significance (*p* = 0.06). Human herpes virus-6 positivity was lower in nasal polyps (13/91; 14.29%) versus controls (10/38; 26.32%, *p* = 0.13). In chronic rhinosinusitis with nasal polyps group, 1 sample was herpes simplex virus-1-positive (1/91; 1.1%), and another was cytomegalovirus-positive (1/91; 1.1%), versus none in controls. No sample was positive for herpes simplex virus-2, varicella-zoster virus, high-risk-human papilloma viruses (16, 18, 31, 33, 35, 39, 45, 51, 52, 56, 58, 59) and low-risk-human papilloma viruses (6, 11).

**Conclusion:**

Differences in Epstein–Barr virus and human herpes virus-6 positivity among patients with chronic rhinosinusitis with nasal polyps and healthy controls are not statistically significant, weakening the likelihood of their implication in chronic rhinosinusitis with nasal polyps pathogenesis.

## Resumo

**Introdução:**

A rinossinusite crônica com pólipos é uma doença multifatorial de etiopatogênese ainda não definida. Existem dados contraditórios na literatura sobre a implicação potencial de elementos virais na etiologia de pólipos nasossinusais.

**Objetivo:**

Comparar a prevalência de herpes vírus humanos (1–6) e papiloma vírus humano em pacientes adultos com rinossinusite crônica com pólipos nasais (CRwNP) e controles saudáveis.

**Método:**

A presença de DNA viral foi avaliada por PCR em tempo real, em amostras de pólipos nasais de 91 pacientes com CRwNP e na mucosa das conchas nasais de 38 controles saudáveis.

**Resultados:**

A positividade do EBV foi maior nos pólipos nasais (24/91; 26,4%) do que nos controles (4/38; 10,5%), mas a diferença não foi significante (p = 0,06). O HHV-6 apresentou positividade menor nos pólipos nasais (13/91; 14,29%) do que os controles (10/38; 26,32%), (p = 0,13). No grupo CRwNP, uma amostra foi positiva para o vírus herpes simples (HSV-1) (1/91; 1,1%), e uma para citomegalovírus (CMV) (1/91; 1,1%); e nenhuma amostra foi positiva no grupo controle. Não houve amostra positiva para HSV-2, VZV, HR-HPV (16,18, 31, 33, 35, 39, 45, 51, 52, 56, 58, 59) e LR-HPV (6,11).

**Conclusão:**

Diferenças de positividade do EBV e HHV-6 entre pacientes com CRwNP e controles saudáveis não são estatisticamente significantes, enfraquecendo a probabilidade de sua implicação na patogênese da CRwNP.

## Introduction

Chronic rhinosinusitis with nasal polyps (CRSwNP) is a subdivision of idiopathic chronic rhinosinusitis (CRS).^1^ It is a clinical syndrome characterized by persistent symptomatic inflammation of the nasal and paranasal sinuses mucosa.^1^ The etiopathogenesis of CRSwNP is mainly attributed to a dysfunctional host–environment interaction.^2^ Even though the identification of exogenous agents driving the secondary inflammatory mechanisms has been a field of extensive research, the potential involvement of viral infection is relatively unstudied.^1^

Herpes simplex virus-1 (HSV-1), herpes simplex virus-2 (HSV-2), varicella-zoster virus (VZV), cytomegalovirus (CMV), Epstein–Barr virus (EBV), and human herpes virus-6 (HHV-6), along with human papilloma viruses (HPV), are DNA viruses that have the capacity to incorporate into host DNA, to establish lifelong latent infections in the upper respiratory mucosa, and to reactivate in immunocompromised conditions.^3^, ^4^, ^5^, ^6^ Only a few studies^7^, ^8^, ^9^, ^10^, ^11^, ^12^, ^13^, ^14^, ^15^, ^16^, ^17^ have investigated their potential role in CRSwNP, while their results are controversial. Furthermore, the highly sensitive quantitative real-time polymerase chain reaction (PCR) technique has been used for detection of these viruses in CRSwNP by only two studies so far.^11^, ^17^

The aims of the present study were to evaluate and compare the prevalence of HHV, high-risk HPV types (HR-HPV; subtypes 16, 18, 31, 33, 35, 39, 45, 51, 52, 56, 58, 59), and low-risk HPV types (LR-HPV; subtypes 6, 11) in nasal tissue samples of patients with CRSwNP and healthy controls by employing the highly sensitive quantitative PCR technique, and to review the related literature.

## Methods

This was a cross-sectional contemporary cohort study, which was conducted prospectively, from January of 2009 to January of 2013, on adult patients with CRSwNP undergoing functional endoscopic sinus surgery (FESS). CRSwNP diagnosis was made according to the criteria of the European Position Paper on Rhinosinusitis and Nasal Polyps (EPOS).^1^ The control group consisted of healthy adult patients with nasal septal deviation undergoing septoplasty without CRS according to EPOS criteria.^1^ Pediatric subjects, as well as patients with asthma, cystic fibrosis, primary ciliary dyskinesia, allergic fungal sinusitis, allergic rhinitis, inverted papilloma, and HIV seropositivity were excluded from the study. Subjects in both groups who had had an upper respiratory tract infection within two weeks before surgery, and those who had taken any nasal or systemic steroids within the last month prior to surgery were excluded from the study.

In CRSwNP patients, nasal polyp specimens were obtained from the paranasal sinuses during FESS, while in the control group tissue biopsies from the inferior turbinate mucosa were taken during septorhinoplasty. Nasal polyps and nasal tissues extracted during surgery were immediately transferred in sterile dry containers and shipped to the laboratory. By use of a surgical knife, the tissues were cut in half, and several pieces (2–4 mm) were taken from the deep tissue and divided into three parts: for conventional culture, for molecular techniques and for storage at −80 °C.

For each patient, the tissue pieces were inoculated on Sabouraud agar at 30 °C for 10 days and then on 5% sheep blood Columbia agar incubated in a 5% CO_2_ atmosphere and an anaerobic atmosphere for 2 days. Gram staining was performed on all specimens in order to evaluate the presence of leukocytes and microbial flora.

Samples of tissue from each patient were chosen for DNA extraction using commercial kits (Invitrogen) according to the manufacturer's instructions. The efficiency of DNA extraction and the possible presence of inhibitors in the sample were confirmed by the detection of the β2-globin gene using the primers RS42 (5′-GCTCACTCAGTG TGGCAAAG-3′) and Km (5′-GGTTGGCCAATCTACTCCCAGG-3′).

The extracted DNA specimens were submitted to quantitative real-time PCR (Applied Biosystems 7500 Fast Real-Time PCR System) by using commercially available assays according to the manufacturer's instructions: HSV1 Q-PCR Alert AmpliMIX, HSV2 Q-PCR Alert AmpliMIX, VZV Q-PCR Alert AmpliMIX, EBV Q-PCR Alert AmpliMIX, Q-CMV Real Time Complete, HHV-6 Q-PCR Alert AmpliMIX (Nanogen Advanced Diagnostics S.r.L), the HPV High Risk Screen Real Time PCR (Sacace Biotechnologies) and HPV 6/11 Real-TM Real Time PCR kit (Sacace Biotechnologies).

All data, including patients’ demographic information (age, gender, history) were placed in a database. For statistical analysis, Fisher's exact test was used. Data analysis was performed with SPSS v. 20 software (IBM, Chicago, IL, United States). *p*-values <0.05 were considered as statistically significant.

The samples were analyzed at the Department of Microbiology, Medical School, University of Thessaly. The study was approved by the institutional review board (approval protocol No. 10/28-11-2007). A written informed consent was obtained from all patients and control subjects.

## Results

There were 129 white subjects enrolled in the study. The nasal polyps group consisted of 91 patients (63 males; mean age 53 years; range 19–77 years) while the control group consisted of 38 subjects (22 males; mean age 43 years; range 18–54 years). Primary surgery was performed in 65 patients and revision surgery in 26 patients (epidemiological data are summarized in Table 1).

**Table 1:** Epidemiological data on sex, age, and type of surgery (primary or revision) for chronic rhinosinusitis with nasal polyps (CRSwNP) and control groups.

	CRSwNP group	Control group
Total	91	38
Male (%)	63 (69.2%)	22 (57.9%)
Female (%)	28 (30.8%)	16 (42.1%)
Mean age (range)	53 (19–77)	43 (18–54)
Primary surgery (%)	65 (71.4%)	N/A
Revision surgery (%)	26 (28.6%)	N/A

Conventional cultures showed that all specimens were negative for fungi, while they were positive for Gram-positive cocci of microbial flora, including *Staphylococcus aureus*, coagulase-negative staphylococci, and viridans streptococci.

DNA extraction, indicated by β2-globin gene detection, was successful in all the samples.

EBV positivity was higher in nasal polyps than the control group (polyps group: 24/91; 26.4% versus control group: 4/38; 10.5%). This difference did not reach significance (*p* = 0.06).

HHV-6 positivity was lower in nasal polyps than the control group (polyps group: 13/91; 14.29% versus control group: 10/38; 26.32%). This difference was also not significant (*p* = 0.13).

One nasal polyp sample (1/91; 1.1%) was found to be HSV-1 positive and one sample (1/91; 1.1%) was found to be CMV positive. All control group samples were negative for the HSV-1 and CMV. These differences were not significant.

In nasal polyps group, the specimens of four patients were positive for both EBV and HHV-6, while in one patient they were positive for both EBV and CMV. Simultaneous positivity in EBV and HHV-6 was also noticed in two subjects of the control group.

All specimens of study and control groups were negative for HSV2, VZV, HR-HPV, and LR-HPV DNA (results are summarized in Table 2).

**Table 2:** Rates of HSV-1, HSV-2, VZV, CMV, EBV, HHV-6, and HPV (HR-HPV and LR-HPV types) in chronic rhinosinusitis with nasal polyps (CRSwNP) and control groups.

	CRSwNP (*n* = 91)	Control (*n* = 38)	Significance^a^
	Positive – *n* (%)	Positive – *n* (%)	
HSV1	1 (1.1%)	0 (0%)	NS
HSV2	0 (0%)	0 (0%)	NS
VZV	0 (0%)	0 (0%)	NS
EBV	24 (26.4%)	4 (10.5%)	NS
CMV	1 (1.1%)	0 (0%)	NS
HHV-6	13 (14.29%)	10 (26.32%)	NS
HR-HPV	0 (0%)	0 (0%)	NS
LR-HPV	0 (0%)	0 (0%)	NS

NS, non-significant; HSV-1, herpes simplex virus-1; HSV-2, herpes simplex virus-2; VZV, varicellazoster virus; CMV, cytomegalovirus; EBV, Epstein–Barr virus; HHV-6, human herpes virus-6; HPV, human papilloma viruses; HR-HPV, high-risk HPV; LR-HPV, low risk HPV.

a Fisher's exact test

## Discussion

Kozak et al.^7^ were the first to investigate the potential role of EBV in the etiology of nasal polyps. In a pilot study on nine patients with CRSwNP and six controls using *in situ* hybridization (ISH), they reported that EBV DNA was absent in both groups. Similar findings with ISH were published by Sham et al.^8^ in 2012, in 30 CRSwNP patients and 32 controls. In contrast, Tao et al.^9^ were the first to report nasal polyp mucosa as one of the sites of EBV persistence. They studied 13 CRSwNP patients and, by using Southern blot hybridazation (SBH), qualitative PCR, and ISH, found EBV positivity in 15%, 69%, and 85%, respectively. However, no controls were used in their study. Zaravinos et al.^10^ compared nasal polyp samples of 23 patients to a control group of 13 inferior turbinate specimens from patients undergoing nasal corrective surgery, by using qualitative PCR. They found EBV positivity in 35% of CRSwNP patients versus 0% in their control group, demonstrating a significant correlation between EBV presence and nasal polyp formation. Recently, Costa et al.^11^ were the first to use quantitative PCR in 35 patients with CRSwNP, in order to compare the occurrence of EBV in nasal polyps and adjacent inferior turbinates tissue samples. They found that EBV positivity tended to be higher in CRSwNP, suggesting a potential causative role or persistence in the inflammatory lymphoid tissue, but this difference did not reach significance. However, the main limitation of their study was the lack of a control group of healthy subjects. The present results are supportive to those published by Costa et al.,^11^ since EBV positivity was higher in CRSwNP than healthy controls, but this difference did not reach significance (*p* = 0.06).

Regarding HPV, controversial findings have also been reported. Bradnsma et al.^12^ and Gaffey et al.,^13^ by using SBH and ISH respectively, reported zero HPV positivity in CRSwNP patients. Becker et al.^14^ and Sham et al.,^8^ by using qualitative PCR, also did not find any HPV positivity in both CRSwNP patients and controls. In 2000, Hoffman et al.^15^ reported a single suspicious HPV positive sample in a group of 33 nasal polyp patients by using SBH and qualitative PCR, while Zaravinos et al.,^10^ utilizing qualitative PCR (GP5+/6+ non-type-specific primers), found a non significant presence of HPV in CRSwNP patients (3/23, 13%) compared to their controls (0/13 inferior turbinates).

On the contrary, Fei Pei et al.,^16^ in a large-scale Chinese study, used qualitative PCR and flow-through hybridization as well as gene chip technology for detection of low risk-HPV (LR-HPV) and HR-HPV in 204 CRSwNP patients and 36 healthy controls (middle turbinate mucosa). They reported 40.2% HPV positivity in CRSwNP versus 0% in controls. In their study, 13 HPV genotypes were found in CRSwNP samples (LR-HPV subtypes: 11, 6, 34, 70, 44; and HR-HPVs subtypes: 58, 52, 18, 16, 68, 53, 31, 33), with LR-HPV-11 the most prevalent (45.28%). The present study did not confirm the results published by Fei Pei et al.^16^ The different findings could be attributed to differences in study populations (Asian versus white) or the method used to detect HPV infection (qualitive versus quantitative PCR). Recently, Rizzo et al.,^17^ in a white population of 20 CRSwNP patients and ten controls, used quantitative real time PCR for the first time with HR-HPV (subtypes: 16, 18, 31, 33, 35, 39, 45, 52, 53, 56, 58, 59, 66, and 70) and LR-HPV (subtypes: 6, 11). No HR-HPV positivity was found in their study. Regarding LR-HPV, they reported a 50% HPV-11 presence in the subgroup of ten CRSwNP patients without allergic disease, while no positive samples were found in the subgroup of ten CRSwNP patients with allergic disease, as well as in their control group. The authors suggested that clinical parameters, such allergy, could be a confounder for the HPV results observed. Furthermore, they suggested that the presence of HPV-11 might be a prognostic marker in the follow-up of CRSwNP without allergic disease, since they noticed that the five HPV-11 positive patients presented with a relapsing nasal polyposis.^17^ In the present study quantitative PCR was used in a large population of 91 CRSwNP patients and 38 controls. These results confirm those published by Rizzo et al.^17^ regarding the absence of HR-HPV in both patients and controls. However, this study did not confirm the high prevalence of LR-HPV-11 reported in their study. The differences between the present findings may be attributed to the differences in the size of the samples. It should be noted that this study did not find any LR-HPV positivity, while allergic patients were excluded, and 26 patients underwent revision surgery.

Zaravinos et al.^10^ were the first to investigate HHV-6 presence in nasal polyp tissue. By using qualitative PCR, they found that HHV-6 DNA was absent in both nasal polyps and inferior turbinate samples of the control group. However, Costa et al.^11^ used quantitative PCR in nasal polyps and inferior turbinate samples of 35 patients with CRSwNP, and found an HHV-6 positivity of 8% in nasal polyps and 35% in adjacent turbinate mucosa. This difference was not significant, and there was no control group of healthy subjects in their study. The present data support the findings of Costa et al.,^11^ since HHV-6 positivity was lower in nasal polyps than inferior turbinate samples of the control group (14.29% versus 26.32% respectively), while this difference was also not significant. It is probable that the difference between the present results and those published by Zaravinos et al.^10^ may be due to the higher sensitivity and specificity of quantitative versus qualitative PCR.^11^

The presence of HSV-1, HSV-2, VZV, and CMV was investigated by Zaravinos et al.^10^ with qualitative PCR and by Costa et al.^11^ with quantitative PCR. Both reported similar findings (two positive HSV-1 samples and one CMV sample each, while no HSV-2 or VZV presence was found) in line with the present data.

The main weakness of this study is that there was a difference in the sample size of the CRSwNP (*n* = 91) and control (*n* = 38) groups. These sample sizes are so different because, within the study period, only 38 of the septoplasty patients accepted to participate. This difference in sample size might have influenced the results and may explain some differences with the results already reported in the literature. Furthermore, as stated above, the results may be different with those already published due to the different methods used to detect the viruses (ISH versus SBH versus qualitative PCR versus quantitative PCR). It should be noticed that apart from this study, only Costa et al.^11^ have used quantitative PCR. This is also one of the strengths of this study, since quantitative PCR is the method with the highest sensitivity and specificity.

## Conclusion

These data demonstrate that EBV and HHV-6 were detected with quantitative PCR in nasal polyps specimens, even though with no significance; moreover, this study showed that HR-HPV and LR-HPV were absent in nasal polyps and controls.

## Conflicts of interest

The authors declare no conflicts of interest.

## References

[1] Fokkens W.J., Lund V.J., Mullol J., Bachert C., Alobid I., Baroody F. (2012). EPOS 2012: European position paper on rhinosinusitis and nasal polyps 2012: a summary for otorhinolaryngologists. Rhinology.

[2] Kern R.C., Conley D.B., Walsh W., Chandra R., Kato A., Tripathi-Peters A. (2008). Perspectives on the etiology of chronic rhinosinusitis: an immune barrier hypothesis. Am J Rhinol.

[3] Maglennon G.A., McIntosh P., Doorbar J. (2011). Persistence of viral DNA in the epithelial basal layer suggests a model for papillomavirus latency following immune regression. Virology.

[4] Doorbar J. (2013). Latent papillomavirus infections and their regulation. Curr Opin Virol.

[5] Efstathiou S., Preston C.M. (2005). Towards an understanding of the molecular basis of herpes simplex virus latency. Virus Res.

[6] Kalla M., Hammerschmidt W. (2012). Human b cells on their route to latent infection-early but transient expression of lytic genes of Epstein-Barr virus. Eur J Cell Biol.

[7] Kozak F.K., Mahony J.B., Chernesky M.A., Newhouse M.T., Dolovich J., Hitch D.A. (1991). Nasal polyposis: in search of a viral etiology using DNA hybridization. J Otolaryngol.

[8] Sham C.L., To K.F., Chan P.K., Lee D.L., Tong M.C., Van Hasselt C.A. (2012). Prevalence of human papillomavirus, Epstein–Barr virus, p21, and p53 expression in sinonasal inverted papilloma, nasal polyp, and hypertrophied turbinate in Hong Kong patients. Head Neck.

[9] Tao Q., Srivastava G., Dickens P., Ho F.C. (1996). Detection of Epstein–Barr virus-infected mucosal lymphocytes in nasal polyps. Am J Pathol.

[10] Zaravinos A., Bizakis J., Spandidos D.A. (2009). Prevalence of human papilloma virus and human herpes virus types 1–7 in human nasal polyposis. J Med Virol.

[11] Costa C., Garzaro M., Boggio V., Sidoti F., Simeone S., Raimondo L. (2014). Detection of herpesviruses 1-6 and community-acquired respiratory viruses in patients with chronic rhinosinusitis with nasal polyposis. Intervirology.

[12] Brandsma J., Sciubba J., Shah K., Barrezueta N., Galli R. (1987). Papilloma infection of the nose. Cancer Cells.

[13] Gaffey M.J., Frierson H.F., Weiss L.M., Barber C.M., Baber G.B., Stoler M.H. (1996). Human papillomavirus and Epstein–Barr virus in sinonasal schneiderian papillomas. An in situ hybridization and polymerase chain reaction study. Am J Clin Pathol.

[14] Becker M., Forslund O., Hansson B.G., Malm L. (1994). Search for the human papillomavirus in nasal polyps, using a polymerase chain reaction-method. J Otolaryngol.

[15] Hoffmann M., Kahn T., Goeroegh T., Lohrey C., Gottschlich S., Meyer J. (2000). Tracing human papillomavirus DNA in nasal polyps by polymerase chain reaction. Acta Otolaryngol.

[16] Pei F., Chen X.P., Zhang Y., Wang Y., Chen Q., Tan X.J. (2011). Human papillomavirus infection in nasal polyps in a Chinese population. J Gen Virol.

[17] Rizzo R., Malagutti N., Bortolotti D., Gentili V., Rotola A., Fainardi E. (2014). Infection and HLA-G molecules in nasal polyposis. J Immunol Res.

